# No difference in clinical outcome and survivorship after total knee arthroplasty with patellar resurfacing and nonresurfacing after minimum 10-year follow-up

**DOI:** 10.1097/MD.0000000000019080

**Published:** 2020-03-13

**Authors:** Bin Feng, Yi Ren, Jin Lin, Jin Jin, Wenwei Qian, Xisheng Weng

**Affiliations:** Department of Orthopaedic Surgery, Peking Union Medical College Hospital, Peking Union Medical College, Beijing, China.

**Keywords:** clinical outcome, patellar nonresurfacing, patellar resurfacing, primary total knee arthroplasty, survivorship analysis

## Abstract

The study aims to evaluate the clinical outcomes and surgery survivorship for over 10 years following patellar resurfacing or nonresurfacing in total knee arthroplasty (TKA) in a cohort of Chinese patients.

From 1998 to 2003, 355 patients underwent primary TKA in our institute. The survivorship of TKA between the patellar resurfacing and nonresurfacing groups and the clinical outcome of Hospital for Special Surgery knee score, Western Ontario and McMaster Universities index score, patellar score, patellar related complications, and radiological results were studied at latest follow-up.

There was no statistically significant difference for the Hospital for Special Surgery score, Western Ontario and McMaster Universities score, and the patellar score between the 2 groups after an average 12.4-year follow-up. Nonresurfacing group had higher anterior knee pain than the resurfacing group (13.2% vs 5.6%). The patients with rheumatoid arthritis had a 2.9-fold higher rate of patellar-related complications than did the patients with osteoarthritis. The 10-year survival rate was not significantly different between the 2 groups both for revision surgery (*P* = .505) and for patellar-related complication (*P* = .194).

There was no significant difference in the long-term clinical outcome and survivorship between patellar resurfacing and nonresurfacing. Patellar nonresurfacing could be advisable during primary TKA for osteoarthritis patients. Selective patellar resurfacing for RA patients could achieve lower patellar-related complications.

## 1 Introduction

Patellar resurfacing has been recommended for anterior knee pain (AKP) associated with the patella after total knee arthroplasty (TKA). However, complications such as polyethylene wearing, patellar fracture, and patellar clunk syndrome might occur after patellar resurfacing.^[1]^ The following 3 approaches are used for the patellar strategy during primary TKA:

1. never resurface the patella;
2. always resurface the patella; and
3. selectively resurface the patella.^[2]^

Whether the patella should be routinely resurfaced during primary TKA is controversial, as is the criteria for resurfacing. A nation-wide survey conducted in the United States in 2012 reported that the patella was resurfaced in 96% of all primary TKA procedures.^[3]^ The number of TKA receiving patellar resurfacing has steadily declined in Sweden according to the Swedish Knee Arthroplasty Register, with the rate of patellar resurfacing being only 3% in 2010.^[4]^ Randomized controlled trials have shown that patellar resurfacing is not statistically different from nonresurfacing regarding the clinical outcome, knee score, and reoperation rate.^[5–7]^ Other studies and meta-analyses have reported that patellar resurfacing might demonstrate superiority.^[8,9]^ It was reported that the knee anatomy of Chinese population was different from that of western populations because of the relatively thinner patella and thinner anterior femoral condyles.^[10]^ Although less patients accept patellar resurfacing during primary TKA in Chinese mainland than in the Western country, there have been limited reports regarding the long-term outcome of patellar resurfacing in Chinese subjects. The purposes of our study were to evaluate the clinical and radiographic outcome after 10 postoperative years between a patellar resurfacing group and nonresurfacing group during primary TKA and the survivorship at more than 10-year follow-up.

## 2 Methods

The study protocol was approved by the institutional review board of Peking Union Medical College Hospital (protocol number: S-K737). Ethics Committee approval was taken from the regional Ethics Committee, and oral informed consent was obtained from all the participants for their clinical records to be used in this study. The study retrospectively enrolled 355 consecutive patients (393 knees) undergoing primary cemented TKAs between 1998 and 2003 in our institute. The inclusion criteria were as follows:

1. the patients underwent primary cemented TKAs;
2. the patients underwent TKAs for knee osteoarthritis (OA) or rheumatoid arthritis (RA).

**Figure 1:**
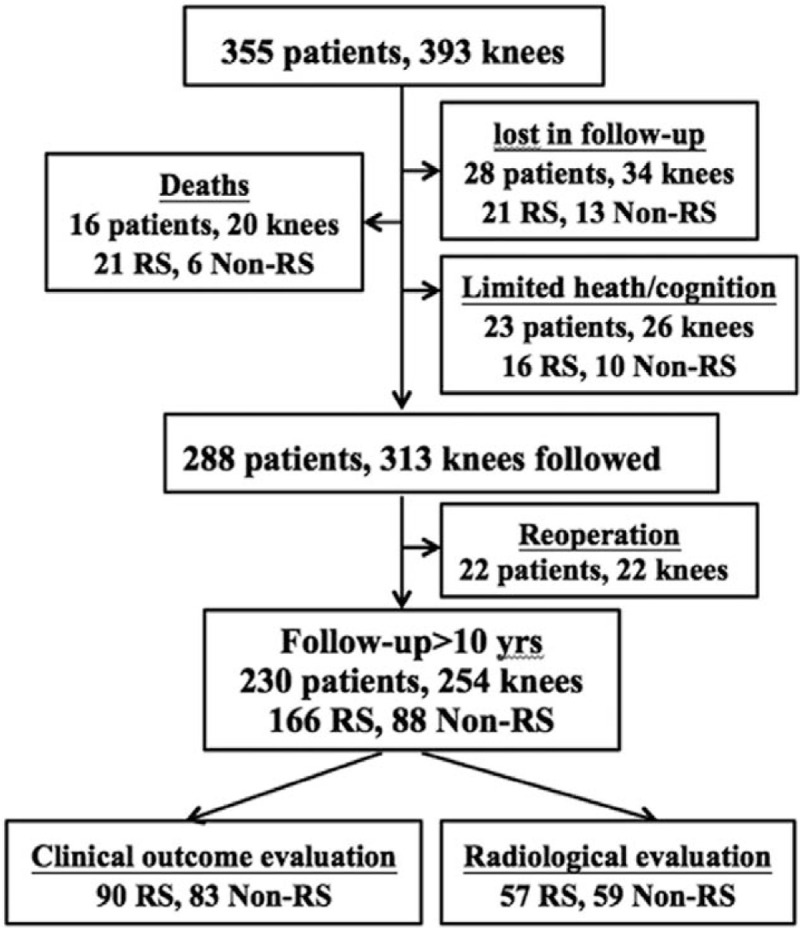
Patient follow-up flowchart. Non-RS = patellar nonresurfacing, RS = patellar resurfacing.

Patients who underwent revision surgery would meet with the exclusion criteria. The indications for surgery were 57 patients with RA and 298 patients with knee OA. Among them, 28 patients (34 knees) were lost during follow-up. Sixteen patients (20 knees) died and 23 patients (26 knees) had limited health/cognition before 10 years postoperatively; all of these patients had TKAs in situ at the latest follow-up. Therefore, the total number of patients who were successfully followed up was 288. Follow-up concluded in December 2015. The overall follow-up rate was 79.6% (313 knees) in our series, with mean follow-up time of 8.76 years (range from 2 to 17 years). Flowchart of the follow-up is provided in Figure 1.

### 2.1 Surgical technique

A medial parapatellar approach to the knee was used. In our institute, the patellar was regularly resurfaced before 2001 unless the patellar was inadequate to be resurfaced because of the inadequate bone quality and thickness of patellar, and the patellar was not resurfaced thereafter because of the upgrade of knowledge. Generally, the residual bone thickness of more than 12 mm was required for patellar resurfacing,^[11]^ and resurfacing with a polyethylene dome patella was done in the resurfacing group in our study. For the patellar nonresurfacing group, patelloplasty consisting of articular surface smoothing, osteophyte removal, and patellar rim denervation was performed. Patellofemoral tracking was assessed after implantation of final implants, and the need for lateral release was assessed.

In this study, the average patella thickness for patients with OA was 21.1 mm (13.5–27.6 mm) in our series, with a mean thickness of 20.9 mm for females and 22.7 mm for males. The thickness for patients with RA was 20.6 mm (13.3–26.1 mm).

### 2.2 Clinical and radiological evaluation

The patients who completed a 10-year follow-up were evaluated. The patients underwent clinical evaluation at the outpatient clinic. In cases in which it was not possible for the patient to visit the outpatient clinic, the patients were visited by an examiner from the local hospital and were interviewed with a telephone questionnaire. The Hospital for Special Surgery (HSS) knee score questionnaire and the Western Ontario and McMaster Universities (WOMAC) Osteoarthritis Index were used for the clinical evaluation. AKP when climbing stairs, range of motion (ROM), patellar clunk, instability, and the reason for revision were recorded at the latest follow-up. The patellar score was evaluated according to the Fellar system.^[12]^ For the patients who had died at the time of the study, the date of death and the status of the knees as well as the knee scores were recorded from the family.

X-ray images were obtained immediately post-surgery, and weight-bearing X-ray examinations in the anteroposterior (AP), laterolateral (LL), and skyline views were evaluated at the latest follow-up. The skyline views of the patella were evaluated for patellar tracking. The Insall–Salvati index was used to evaluate the relationship of the patella with the joint line.

### 2.3 Statistical analysis

SPSS (SPSS, Inc, Chicago, IL) was used for the statistical analysis. The clinical data were analyzed using the means and standard deviations. The level of statistical significance was set at *P* < .05. Independent 2-sided paired *t*-tests were performed to determine the difference in the HSS knee score, WOMAC score, and the ROM. For categorical variables, chi-square analysis was used to compare the difference. The Kaplan–Meier method was used for the survivorship analysis. For the patients who could not complete the clinical evaluation during the latest follow-up, we presumed that the implants worked well in situ if no problems with the TKA were described at the last visit. The survivorship between different factors was evaluated in a log rank test. The risk factor of patellar related complications was analyzed with Cox proportional hazards model. Hazard ratio (HR) with 95% confidence interval (CI) was used to evaluate the relative risk between the different groups. A 2-tailed post hoc power analysis was conducted to detect a 10% difference in clinical outcome assuming an α error of 5% by using the software G∗Power (version 3.1.9.2, Franz Faul, University of Kiel, Kiel, Germany).

## 3 Results

In our series, 288 patients (313 knees) were successfully followed up. The demographic information is provided in Table 1. No significant differences were found between the resurfacing and nonresurfacing groups regarding age, sex, body mass index, diagnosis, and posterior cruciate ligament strategy. A total of 230 patients (254 knees) presented implants with good performance in situ after more than 10 postoperative years, with an average follow-up of 12.4 years (10–19 years) (Fig. 1). The indications for TKA were 46 knees for RA and 208 knees for primary or secondary OA. The Ortholoc knee system (Dow Corning Wright Medical, Arlington, TN) was used for 82 knees. PFC knee system (DePuy Orthopaedics, Inc, Warsaw, IN) was used for 59 knees. Kinemax knee system (Howmedica, Rutherford, NJ) was used for 98 knees. Domestic knee system (Montagne, Beijing, China) was used for others.

**Table 1:**
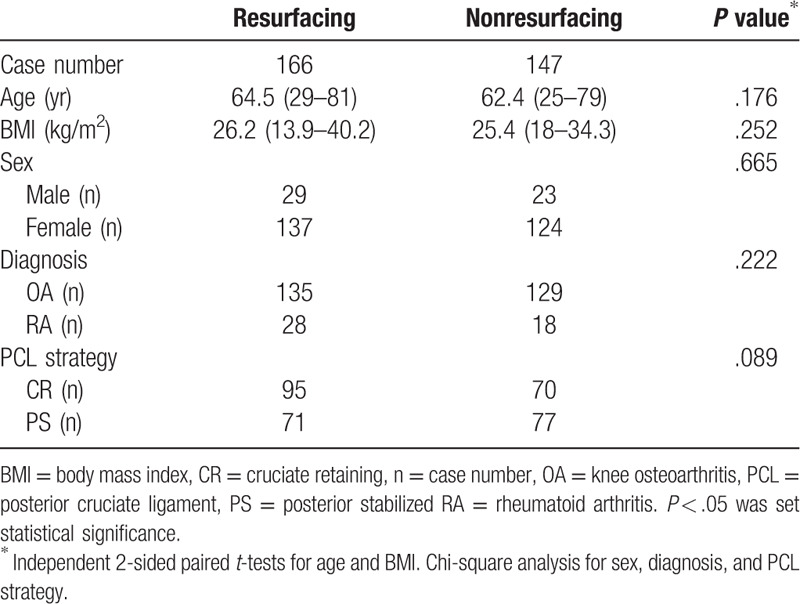
Demographic information of the knees successfully followed-up after primary total knee arthroplasty.

### 3.1 Clinical results

The clinical outcomes were successfully evaluated for 173 knees with an HSS knee score, patellar score, and WOMAC score (Table 2). Post hoc power analysis showed >80% statistical power to detect a 10% difference in HSS score, postoperative ROM, patellar score of the 2 groups, and 98% statistical power to detect a difference in postoperative ratio of AKP (Table 2). However, our study was underpowered to make conclusions regarding WOMAC score and ratio of patellar complication (Table 2). The total HSS knee score increased from 55.66 ± 12.8 (range: 28––87) preoperatively to 92.04 ± 8.67 (range: 72–96) postoperatively. The ROM improved from 85.7° ± 25.5° (range: 10°–120°) to 98.2° ± 14.3° (range: 30°–125°). All the items had statistically significant differences (*P* < .01). In our series, there was no significantly different clinical outcome improvement between the patellar resurfacing and patellar nonresurfacing groups according to the HSS knee score, the patellar score, the WOMAC score, and the ROM (Table 2).

**Table 2:**
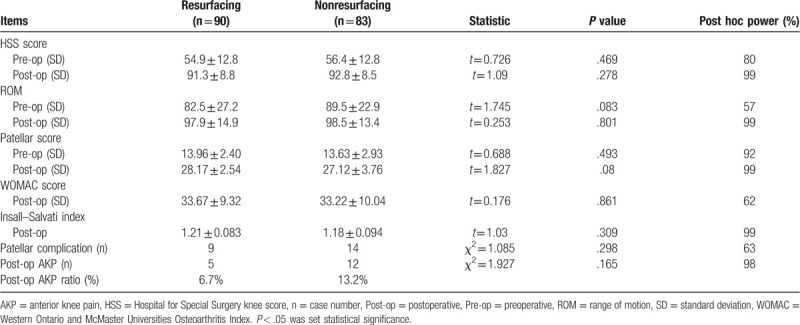
Long-term follow-up clinical evaluation with HSS knee score, patellar score, WOMAC score, ROM, complication due to patellar, and AKP in knees underwent total knee arthroplasty with patellar resurfacing and nonresurfacing.

In our study, a total of 22 patients (22 knees) underwent reoperation during follow-up (Fig. 1). Four patients underwent reoperations for patellar complication, consisting of 1 patellar dislocation from patellar resurfacing group 9 years after the index operation, 1 patellar fracture from patellar resurfacing group 2 years after the index operation, and 2 from patellar nonresurfacing group who underwent revision to resurfacing because of AKP 1 year after the index operation.

Nine patients experienced patellar complications in the resurfacing group, with 1 patient presenting with a patellar dislocation, 1 patient with patellar fracture, 5 patients with AKP, and 2 patients with patellar clunk. Of the 13 patients who experienced patellar complications in the nonresurfacing group, 2 patients had patellar clunk and 11 patients had AKP. Only 2 patients in nonresurfacing group accepted reoperation for severe AKP at postoperative 1 year. The patellar nonresurfacing group experienced a higher rate of AKP compared with the resurfacing group (13.2% vs 5.6%). The difference was not statistically significant for patellar complication (χ^2^ = 1.247, *P* = .264) and AKP (χ^2^ = 3.048, *P* = .081) between the 2 groups (Table 2). The difference was not statistically significant for patellar complication between OA and RA patients after patellar resurfacing (χ^2^ = 2.426, *P* = .121), whereas the difference was significant after patellar nonresurfacing (χ^2^ = 4.205, *P* = .037) (Table 3).

**Table 3:**
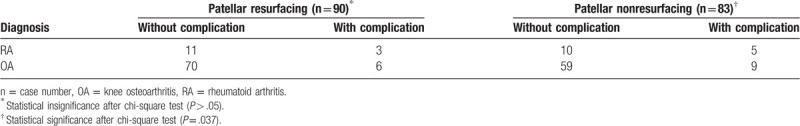
Patellar-related complications between RA and OA patients for different patellar strategy.

According to Cox proportional hazards model, there was a significant difference in patellar-related complications between the RA and OA patients (*P* = .016), whereas there was no significant difference in patellar-related complications between the patellar resurfacing and nonresurfacing patients (*P* = .066) or between the cruciate retaining and posterior stabilized patients (*P* = .203). The RA patients experienced a 2.9-fold higher incidence of patellar-related complications compared with the OA patients (HR = 2.9, 95% CI 1.23–6.59). Patellar nonresurfacing experienced a 2.2-fold higher incidence of patellar-related complications compared with the resurfacing patients (HR = 2.2, 95% CI 0.95–5.07).

### 3.2 Radiological results

Radiological evaluation was possible for 116 knees in our study. The Insall–Salvati index was 1.21 (range: 0.8–1.4) for the resurfacing group and 1.18 (range: 0.8–1.43) for the nonresurfacing group (Table 2). In the cases in which the edge of the patella presented with lateralization over the edge of the lateral condyle on the skyline view, patellar lateral subluxation was considered. The chi-square test showed that the nonresurfacing group had a higher and statistically significant rate of lateral subluxation at follow-up than did the resurfacing group (χ^2^ = 4.265, *P* = .039) (Table 4).

**Table 4:**
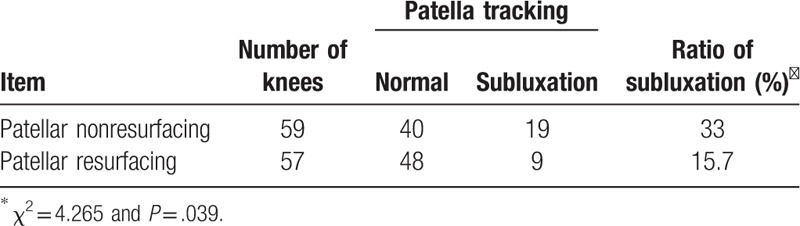
Patellar tracking between patellar resurfacing group and nonresurfacing group.

### 3.3 Survivorship analysis

**Figure 2:**
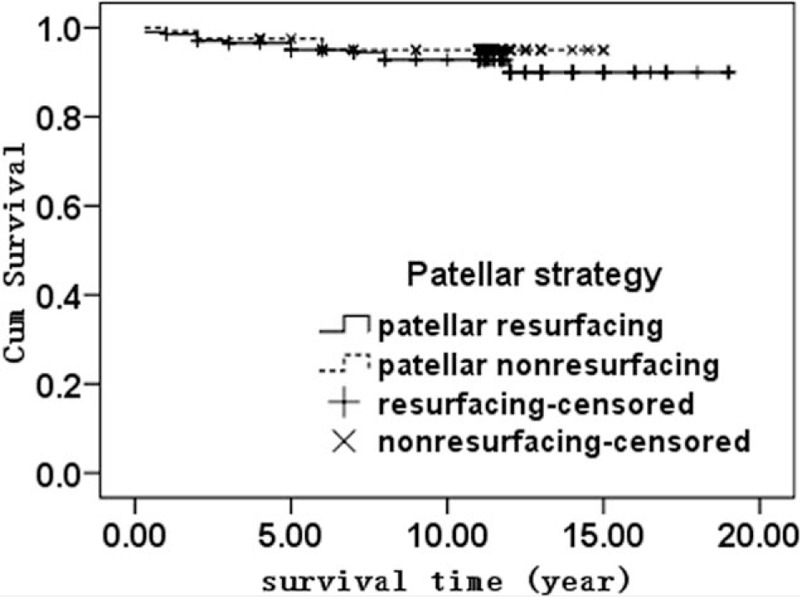
The Kaplan–Meier survival analysis of TKA with a reoperation for the implant as the endpoint between the patellar resurfacing group and the nonresurfacing group (*P* = .34).

**Figure 3:**
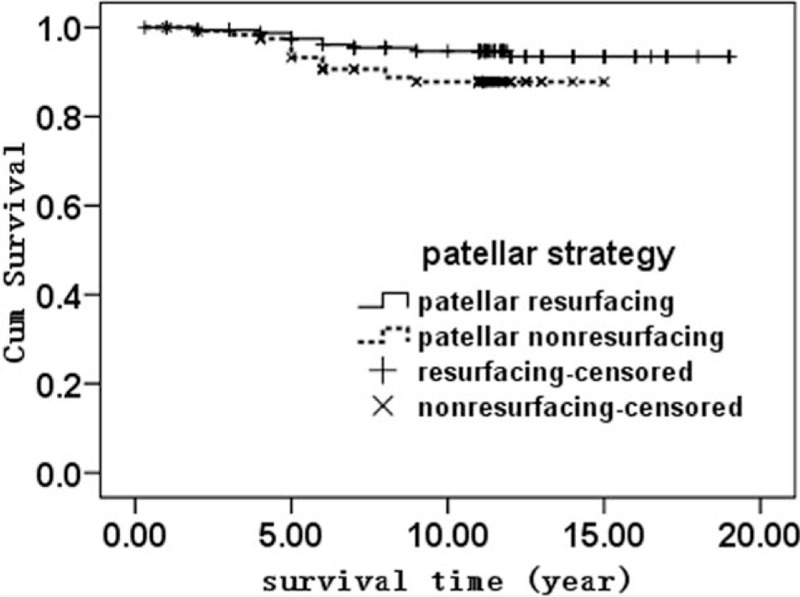
The Kaplan–Meier survival analysis of TKA with patellar complications as the endpoint between the patellar resurfacing group and the nonresurfacing group (*P* = .061).

In our cohort, taking failure as reoperation of the implant as the endpoint, the cumulative average survival rate at 10 years was 93.6% ± 1.7%, with 92.8% ± 1.9% for the resurfacing group and 94.2% ± 2.1% for the nonresurfacing group. The difference was not statistically significant (*P* = .505) (Fig. 2). Taking failure as a patellar-related complication, the cumulative average survival rate at 10 years was 92.3% ± 2.6% for the patellar resurfacing group and 86.4% ± 3.5% for the nonresurfacing group. The difference was not statistically significant (*P* = .194) (Fig. 3).

## 4 Discussion

TKA is a highly effective procedure that provides reliable relief from pain and improves the physical function in patients with advanced knee arthropathy. Although long-term follow-up study has been reported for western Caucasians and Asians with good survivorship and improved clinical outcome,^[13,14]^ whether the patella should be routinely resurfaced during primary TKA and the criteria for resurfacing remain controversial.^[3,4]^

A main concern against patellar nonresurfacing is the possibility of postoperative AKP that may require reoperation. According to the literatures, patients whose patellar was not resurfaced during index TKA tended to have a higher revision rate.^[15,16]^ Nevertheless, there are many causes of AKP other than patellofemoral joint,^[17]^ including muscle strength imbalance, inappropriate trochlear design, soft tissue irritation, and psychological factors modifying pain sensation.^[18–21]^ Therefore, secondary patellar resurfacing for AKP in nonresurfaced patellar does not necessarily result in the relieve of the pain.^[15,22,23]^ For post-TKA AKP with patellar nonresurfacing, revision to patellar resurfacing maybe an available option even if it does not lead to resolution of the AKP symptom. And this may account for the greater prevalence of revision for patellar nonresurfacing group.^[15,16]^ Li et al^[24]^ conducted a systematic review of patellar resurfacing in TKA and demonstrated that the average incidence of AKP of the nonresurfacing group and resurfacing group was 24.1% and 12.9%, respectively; the study reported that patellar resurfacing in TKA could reduce the risk of reoperation with no benefit to the postoperative knee function or patient satisfaction than in TKA without patellar resurfacing.^[24]^ The authors of other studies concluded that although the nonresurfacing group had more cases of revision because of a patellar cause, there was no difference in the clinical outcome between different patellar strategies for OA patients.^[5,7,10,17]^

The current study demonstrated that, although, the nonresurfacing group had a higher rate of AKP (13.2% vs 5.2%) and higher rate of postoperative subluxation, the clinical outcome evaluations with the HSS knee score, the patellar score, AKP, patellar complications, and the postoperative WOMAC score were not statistically significantly different after more than 10 years of follow-up between the patellar resurfacing and nonresurfacing groups. Our result was consistent with studies of the outcome of patellar resurfacing after TKA.^[5,7,10,24,25]^ This study further indicated the long-term survivorship was not significantly different between the 2 groups both for revision surgery and patellar-related complications. Regarding the studies, more knee surgeons have concluded that it is not necessary on a regular basis to resurface the patella during primary TKA.^[7,10,12,26]^

In our study, we further analyzed the patellar resurfacing/nonresurfacing in RA patients. We found the diagnosis of RA was more significant factor that affected patellar complications than OA, according to the Cox hazard model (relative risk = 2.9). Patellar resurfacing was considered to eliminate the reaction between the patellar cartilage and inflamed synovium and to reduce postoperative patellar-related complications.^[17,27,28]^ It has been reported that RA patients with TKA and patellar resurfacing were more satisfied with the clinical outcome and had a lower rate of AKP than patients without resurfacing.^[28,29]^ However, the RA patients were reported to have a high incidence of osteopenia and a small patella.^[30]^ The current study also demonstrated the average patellar thickness for RA patients was 20.7 mm. The aforementioned factors resulted in the increased risks of and difficulty for patellar resurfacing for RA patients. So, although the RA could be an indication of patellar resurfacing during TKA, with lower postoperative complications, the knee surgeon should refer to the patellar thickness, bone quality, and patellar deformity after intraoperative inspection as the selection criteria regarding patellar resurfacing. Patellar resurfacing is not advisable in cases in which RA patients have little or no patellar deformity.^[30]^

The most frequently adopted criteria for patellar resurfacing in the literature were the presence of advanced patellofemoral arthritis, damage to the cartilage of the patellar joint surface, and patellofemoral incongruency.^[17,27,29]^ Other criteria included inflammatory OA, preoperative AKP, and advanced deformity.^[17,27]^ For patients presenting with a small and osteopenic patella and active and young patients with moderate damage to the patellar cartilage, patellar resurfacing was not recommended.^[17]^ The average patella thickness was 20.9 mm for females and 22.7 mm for male in our series, which was less than that found in the studies on patients in Western countries, which was 22.5 mm for females and 25.3 mm for males.^[31]^ With the morphological features of the patellar and relatively thinner anterior femoral condyle in a Chinese population^[10]^ and the patella remodeling over the years to match the condylar design in nonresurfacing cases,^[32]^ the pressure in the patellofemoral joint in Chinese patients maybe lower. Furthermore, the denervation may be as effective as patellar resurfacing for the relief of the postoperative AKP.^[33]^ All the above points might indicate the reason for the lower AKP rate in our study than in other studies.^[5,24,29,33,34]^

The present study has several limitations. First, this study was a retrospective study, which risks low data homogeneity and integrity compared with prospective study. Our study was underpowered to detect potential differences of WOMAC and patellar complications between patellar resurfacing and nonresurfacing, which could mean that we missed a clinically difference between the 2 groups. However, our study was sufficiently powered to detect HHS score and ROM between the 2 groups. Our study could serve as preliminary study to further study and for systematic reviews. Second, there was a relatively higher rate of lost to follow-up. Questionnaires from the family of dead patients involved in our study might also give rise to inaccuracy. Those confounding factors would cause biases. However, long-term results of more than 10 years are generally difficult to obtain in a prospective manner, especially among a patient collective that is rather advanced in age. Furthermore, the shortcomings of the aforementioned limitation may be compensated by the long follow-up period. Third, we did not have information about the severity of the intraoperative patellar articular cartilage, and there may be some selection bias as for the different severity of preoperative patellofemoral arthritis between patellar resurfacing and nonresurfacing groups. However, there was no difference as for the preoperative symptoms, knee score, and patellar score between the 2 groups. We concluded the 2 groups in this study were still comparable. Fourth, we used multiple implants design in this study. Although all the implants adopted patella-friendly design, there was underlying different philosophy between different products, which may further result in the study bias. Because of the limitation of the study, further long-term follow-up of modern prostheses in randomized studies will be needed in future study.

## 5 Conclusions

According to our study, we conclude that there was no difference between patellar resurfacing and nonresurfacing in the long-term clinical outcome and survivorship in Chinese patients. Patellar nonresurfacing could be advisable during primary TKA, particularly in OA patients. Selective patellar resurfacing was recommended in TKA for RA patients with a lower rate of patellar-related complications.

## Acknowledgments

The authors are grateful to Dr Yanyan Bian and Lijuan Zhao for the data collection in the study. The authors also are grateful to Leyna D from American Journal Experts for their editing service of the manuscript.

## Author contributions

**Conceptualization:** Bin Feng, Yi Ren, Xisheng Weng.

**Data curation:** Bin Feng, Yi Ren, Xisheng Weng.

**Formal analysis:** Bin Feng, Xisheng Weng.

**Funding acquisition:** Bin Feng, Xisheng Weng.

**Investigation:** Yi Ren, Xisheng Weng.

**Methodology:** Yi Ren.

**Project administration:** Jin Lin, Jin Jin, Wenwei Qian.

**Resources:** Yi Ren, Jin Lin, Jin Jin, Wenwei Qian.

**Software:** Jin Lin, Jin Jin, Wenwei Qian.

**Supervision:** Jin Lin, Jin Jin, Wenwei Qian.

**Validation:** Yi Ren.

**Visualization:** Bin Feng, Yi Ren, Xisheng Weng.

**Writing – original draft:** Bin Feng, Yi Ren, Xisheng Weng.

**Writing – review & editing:** Bin Feng, Xisheng Weng.
